# Emergency Psychiatry Demand and Clinical Outcomes During On-Call Duty: Mixed Method Survey at a Tertiary Care Hospital in Dublin

**DOI:** 10.1192/j.eurpsy.2026.11326

**Published:** 2026-07-31

**Authors:** U. Usman, K. Keenan, A. Moran

**Affiliations:** 1 Department of Liaison Psychiatry, CHI at Temple Street University Hospital, Dublin 1; 2 Associate of the Dean Clinic, St Patrick’s Mental Health Service, Dublin 8, Ireland

## Abstract

**Introduction:**

Psychiatry on-call services play a crucial interface role between emergency and inpatient care. Increasing clinical acuity and emergency department (ED) pressures in Irish hospitals highlight the the importance of contemporary operational data to guide safe staffing and resource planning. However, prospective reporting on psychiatry on-call workload in Irish general hospitals remains limited.

**Objectives:**

To characterise emergency demand, clinical outcomes and Mental Health Act (MHA) use during psychiatry on-call shifts at a Tertiary Care Hospital in Dublin.

**Methods:**

A prospective mixed-method opt-in post-shift survey was completed by on-call non-consultant hospital doctors between July and September 2022. Data recorded included ED referrals received after working hours, assessments completed, patients un-assessable due to intoxication/sedation, working diagnoses, direct admissions, special-population encounters, MHA detentions, restraint/seclusion, self-harm incidents in the Department of Psychiatry (DOP) and liaison general ward reviews. Descriptive analysis was performed.

**Results:**

Of 90 on-call shifts, 62 responses were obtained (68.8%). Across these 62 shifts, 212 ED referrals were received; 76.27% were assessed and 23.73% were unassessable due to intoxication or sedation. Common presentations included depressive episodes (16.03%), alcohol/substance-related presentations (18.86%), and suicide attempts (10.37%). MHA detention was initiated in 15.25% of presentations. Direct admissions to psychiatric ward occurred during 39.29% of shifts. Physical restraint occurred in 18.97% and seclusion in 22.41% of shifts. Self-harm in the DOP was reported in 15.52% of shifts. Inter-hospital transfer requests occurred in 34.48%, and liaison reviews on medical/surgical wards in 20.69%.

**Conclusions:**

High acute workload after working hours, frequent complex presentations, and associated safety interventions were observed. Intoxication was a frequent barrier to timely assessment. The findings support the need for sustained investment in emergency mental health pathways, consideration for extended liaison psychiatry provision, particularly in urban Irish general hospital settings.

**Disclosure of Interest:**

None Declared

